# Whole-exome sequencing of pathogenic genes in a family with congenital heart disease: A case report

**DOI:** 10.1097/MD.0000000000036977

**Published:** 2023-02-02

**Authors:** Li Chang, Renhui Ji, Rina Sa, Jiletu Huge, Caiyan An

**Affiliations:** a Department of Pathophysiology, Basic Medicine College of Inner Mongolia Medical University, Hohhot, China; b Rehabilitation Department, Ordos Central Hospital, Ordos, China; c Department of Pediatrics, Ordos Central Hospital, Ordos, China; d Foundational and Translational Medical Research Center, Department of Allergy and General Surgery, Hohhot First Hospital, Hohhot, China.

**Keywords:** *AMER1* gene, case report, congenital heart disease, *KCNE1* gene, whole-exome sequencing

## Abstract

**Rationale:**

Congenital heart disease (CHD) is the most common birth defect and an important cause of noninfectious deaths in infants and children. It has high prevalence globally, placing an enormous burden on society and families. Studies of individuals with hereditary or sporadic CHD have provided strong evidence for its genetic basis. The aim of this study was to identify causative gene variants in a Chinese family with congenital heart disease.

**Patient concerns and diagnoses:**

Three generations of a CHD family were recruited. Proband III.9 was diagnosed with congenital heart disease at age 11 months, and the echocardiogram showed arterial ductus arteriosus, with a left-to-right shunt at the level of the arteries. Precedent III.10 was a twin of Proband III.9 who was diagnosed with congenital heart disease at age 11 months, in whom the echocardiogram revealed an arterial ductus arteriosus, an unenclosed patent ductus arteriosus, and a left to right shunt at the level of the arteries (second figure). III.8 was diagnosed with congenital heart disease at age 15, but echocardiography in this study showed no abnormalities. No cardiac abnormalities were detected in any of his parents, grandparents, or maternal grandparents. We performed whole-exome sequencing on CHD sufferers and their unexpressing family members to investigate the genetic causes of CHD in this family line. Exome sequencing identified 4 mutation sites in this family line. The variant c.3245A>G (p.His1082Arg) of the *AMER1* gene was consistent with concomitant X-chromosome recessive inheritance, the variant c.238G>C (p.Val80Leu) of the *KCNE1* gene was consistent with autosomal accessory inheritance, and the other 2 variants did not conform to the law of the mode of inheritance of the disease.

**Outcomes:**

The first identified variant, c.3245A>G (p.His1082Arg) of the *AMER1* gene, with X-chromosome recessive inheritance, and the variant c.238G>C (p.Val80Leu) of the *KCNE1* gene, which has been reported as autosomal dominant, may be the causative agent of CHD in this family line. These findings broaden the genetic scope of congenital heart disease and could help in the development of targeted drugs for the treatment of congenital heart disease.

## 1. Introduction

Congenital heart disease (CHD) is a malformation resulting from abnormal development of the heart and great vessels during embryonic life.^[1]^ CHD includes malformations of the heart wall, valves, and blood vessels. The prevalence of CHD as the most common human birth abnormality is 0.8% to 1.2%.^[2]^ Epidemiologic surveys have shown that the incidence of CHD is increasing annually in China.^[3]^ Despite advances in medical care, CHD remains the leading cause of death in infants and children in both developed and developing countries.^[4]^ The causes of CHD are intricate and complex. Although scientists have conducted in-depth research on the pathogenesis of CHD, only 20% of the genes responsible for CHD have been identified, and most CHD is still unexplained, which has greatly limited the development of clinical treatments for CHD.

Over the past decades, several studies have suggested that genetic factors play an important role in CHD.^[5–8]^ Thanks to findings from animal experiments and medical genetics, about 400 genes have been linked to the development of CHD. Disorders associated with the *AMER1* gene and the *KCNE1* gene can partially exhibit a CHD phenotype. The *AMER1* gene is located at human chromosome Xq11.2, with a total length of 3408 bp, encoding APC membrane recruitment protein 1 (AMER1), with a total length of 1135 AA. According to the Online Mendelian Inheritance in Man database, mutations in *AMER1* cause X-linked dominant osteopathia striata with cranial sclerosis (OS-CS).^[9]^ OSCS is a rare X-linked skeletal dysplasia typically associated with longitudinal striae in the metaphyseal region of the long bones and thickening of the skull, as well as macrosomia, dysmorphic facial features, and cardiac septal defects. The relevance of the *AMER1* gene to CHD is that it acts mainly through negative regulation of the Wnt signaling pathway.^[10]^ The *KCNE1* gene is located at human chromosome 21q22.1–21q22.2, and the encoded product is the 13th subunit of the potassium ion channel (Mink protein), a single-transmembrane protein. Mutations in the *KCNE1* gene are the most common cause of congenital defects in long QT syndrome,^[11]^ a heart condition. Variant c.238G > C (p.Val80Leu) of the *KCNE1* gene has been associated with long QT syndrome.^[12,13]^ KCNE1 regulates the potassium voltage-gated channel subfamily Q member 1 (KCNQ1) channel in a variety of ways^[14]^: by slowing voltage-stimulated channel activation, increasing conductance, and eliminating channel inactivation.^[15]^ KCNQ1, a voltage-gated potassium channel composed of 676 amino acids, is essential for cardiac action potential repolarization.

In 1 Chinese family with frequent CHD, we performed whole-exome sequencing of preexisting and unaffected family members. Our analyses using bioinformatics, online software prediction, and genetic pattern analysis, we concluded that variant c.3245A>G (p.His1082Arg) of the *AMER1* gene, which is associated with X-chromosome recessive inheritance, and c.238G>C (p.Val80Leu) of the *KCNE1* gene, which is inherited in an autosomal accessory manner, may cause the CHD in this family line. And the *AMER1* c.3245A>G was discovered for the first time. This enlarges the genetic spectrum of congenital heart disease and provides new ideas for its diagnosis and treatment.

## 2. Case presentation

**Figure 1.**
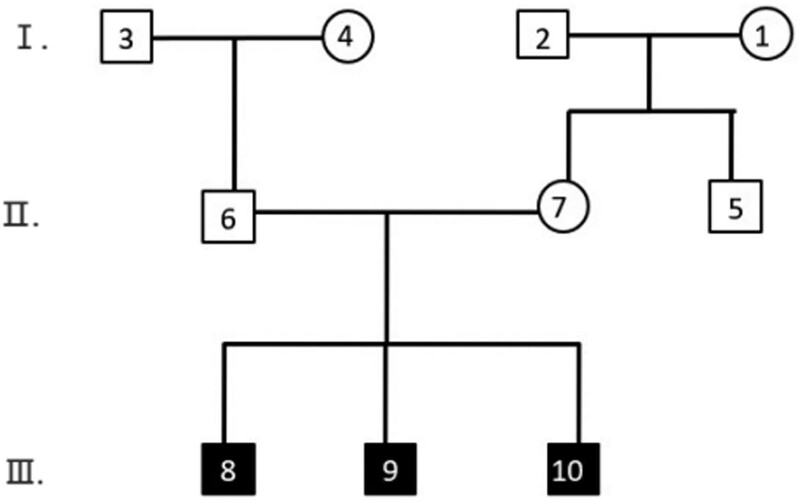
Genealogical map of the family lineage of congenital heart disease. Boxes are males, circles are females, and black represents patients with congenital heart disease.

**Figure 2.**
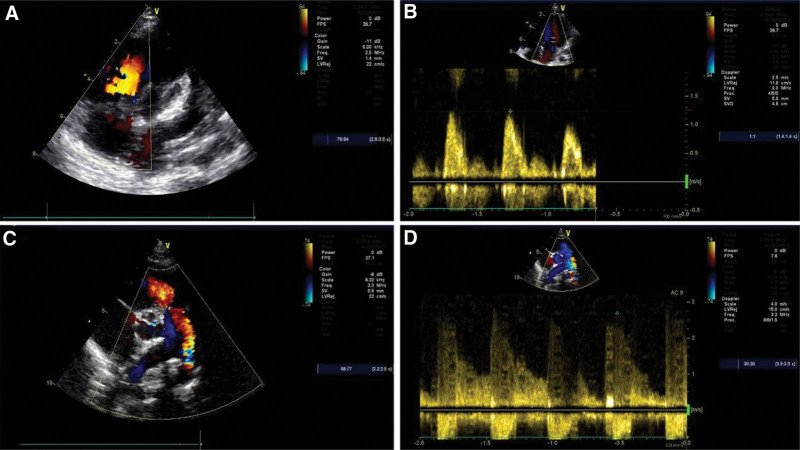
Ultrasonographic findings of the twin probands in this family line. (A and B) Proband III.9, ultrasonographic images showed an unobstructed arterial duct with a left-to-right shunt at the level of the arteries, which was diagnosed as CHD. (C and D) Proband III.10, ultrasonographic images showed an unobstructed arterial duct with a left-to-right shunt at the level of the arteries, which was diagnosed as CHD.

A three-generation CHD Han Chinese family line was recruited from Ordos Central Hospital. All family members were clinically evaluated by reviewing their medical history, performing a physical examination, and reviewing their medical records. This family line consisted of 3 generations totaling 10 individuals (Fig. 1). All participants underwent a detailed physical examination, including auscultation of the precordial region, checking for the presence of other clinical signs and symptoms associated with CHD, and cardiac ultrasound to confirm or exclude the diagnosis of CHD. Proband III.9 was diagnosed with congenital heart disease at age 11 months, and the echocardiogram showed arterial ductus arteriosus, with a left-to-right shunt at the level of the arteries. Precedent III.10 was a twin of Proband III.9 who was diagnosed with congenital heart disease at age 11 months, in whom the echocardiogram revealed an arterial ductus arteriosus, an unenclosed patent ductus arteriosus, and a left to right shunt at the level of the arteries (Fig. 2). III.8 was diagnosed with congenital heart disease at age 15, but echocardiography in this study showed no abnormalities. No cardiac abnormalities were detected in any of his parents, grandparents, or maternal grandparents. Peripheral blood samples were collected from patients and their family members. The study protocol was approved by the Medical Ethics Committee of the First Hospital of Hohhot. Written informed consent was obtained from all participants.

## 3. Whole-exome sequencing and bioinformatics analysis

*Blood sample collection and DNA extraction*: Subjects were fasted overnight, 2 mL of peripheral venous blood was drawn, blood specimens were treated with EDTA for anticoagulation, blood was centrifuged at 3000 r/min for 10 minutes, and then the genomic DNA of the blood samples was extracted with the Bomade Whole Blood Genome Extraction Kit. The concentration and purity of the DNA were determined using a DNA concentration detector (NANODROP2000, Thermo), and the DNA was preserved at −80 °C.

*Whole-exome sequencing*: Whole-exome sequencing of the congenital family line was completed at the Huada Medicine Laboratory in Shenzhen, China. The genomic DNA of the subject’s blood was first broken up, and libraries were prepared. Then, the DNA of the target exons and adjacent shear regions were captured and enriched by the Roche KAPA HyperExome chip. Finally, the MGISEQ-2000 sequencing platform was used for variant detection. The quality control index of the sequencing data was as follows: the average sequencing depth of the target region was ≥180×, among which the proportion of loci with an average depth > 20× in the target region was >95%. Sequenced fragments were aligned with the UCSC hg19 human reference genome (http://genome.ucsc.edu/) by the Burrows–Wheeler Aligner tool (BWA, version 0.7.15) to remove duplicates. Base mass value correction for SNV, INDEL and genotype detection was performed using the Genome Analysis Toolkit (GATK, version 3.3.0). ExomeDepth was used for copy number variation detection at the exon level. Variants with a minimum gene frequency greater than or equal to 1% in databases such as Thousand Genomes (Phase3), ESP6500 (V2), ExAC (r0.3.1), GnomAD (r2.0.1), GnomAD-EAS, etc., were excluded.

*Bioinformatics analysis*: Prediction of nonsynonymous SNPs was performed by SIFT, MutationTaster, and Condel, and the effect of splice site variants on splicing was predicted by SpliceAI (1.3), dbscSNV_ ADA, and dbscSNV_ RF. Nucleic acid conservation was predicted by PhyloP Vertebrates, PhyloP Placetal Mammals, and GERP++. Variant pathogenicity was classified according to the American College of Medical Genetics and Genomics, and American Molecular Pathology Society guidelines for interpretation of sequence variants, with reference to the ClinGen Working Group on Interpretation of Sequence Variants and the British Academy of Clinical Genome Sciences for a refined interpretation of the guidelines.

**Figure 3.**
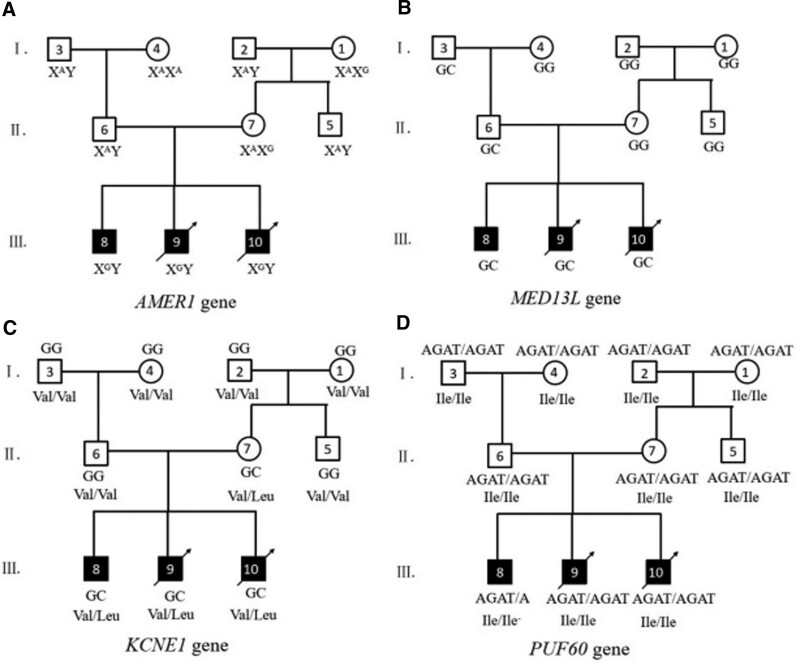
Four genes identified by whole-exome sequencing that may be associated with CHD and the genotypes of the variants carried by each family member, as well as the corresponding amino acid changes.

By exome sequencing, we found 4 mutation sites (Table 1): poly(U) binding splicing factor 60 (*PUF60*) c.1492_1494del ATC(p.Ile498del), *AMER1* c.3245A>G(p.His1082Arg), *KCNE1* c.238G>C(p.Val80Leu), and mediator complex subunit 13L (*MED13L*) c.42G>C(p.[Leu14=]). Irrelevant or meaningless variants, according to multiple bioinformatics analyses, were excluded from the sequencing results. We suggest that the *AMER1* gene variant c.3245A>G (p.His1082Arg), which is accompanied by X-chromosome recessive inheritance, and the *KCNE1* gene variant c.238G>C (p.Val80Leu), which is inherited in an autosomal submissive fashion, are the genetic causes of the family’s CHD (Fig. 3).

**Table 1:** Findings of whole-exome sequencing.

Gene	Location	AA change	SNP	Pathogenicity grade	Genotype	Related diseases and genetic patterns
*PUF60*	chr8: 144898876- 144898878	c.1492_1494delATC(p.Ile4 98del)	rs1064794916	Likely Pathogenic	P: Heterozygous F, M, B: Wild	Verheij syndrome/AD Prenatal phenotypic abnormality/AD
*AMER1*	chrX:63409 922	c.3245A>G (p.His1082Arg)	None	Uncertain Significance	P, B: Hemizygous F: Wild M: Heterozygosity	Prenatal phenotypic abnormality/XL Striped osteopathy with cranial sclerosis/XL
*KCNE1*	chr21:3582 1695	c.238G>C (p.Val80Leu)	rs769368494	Uncertain Significance	P, M, B: Heterozygous F: Wild	Long QT syndrome type 5/AD Jervell and Lange-Nielsen syndrome type 2/AR
*MED13L*	chr12:1167 14895	c.42G>C (p.(Leu14=))	rs141818426	Uncertain Significance	P, F, B: Heterozygous M: Wild	Ectopic right circumflex artery type 1/AD Intellectual impairment and typical facial features with or without heart defects/AD

AA = amino acid, AD = autosomal dominant inheritance, AR = autosomal recessive inheritance, B = brother, F = father, M = mother, P = proband, XL = X-linked inheritance.

The probands, as well as their mother and grandmother, carried a variant in the *AMER1* gene, which was not found in any other family member (Fig. 3A). The analysis of the gene for this variant based on its position on the chromosome indicated that the inheritance of this variant conformed to the X-chromosome recessive mode of inheritance, the probands all being hemizygous, the mother and grandmother heterozygous, and the rest of the family members wild-type, which is in accordance with the law of the mode of inheritance of the disease. A variant in the *KCNE1* gene was found in the mother of the probands, but she did not have the disease (Fig. 3C), while all 3 children carrying the same genotype had the disease, suggesting that the variant was a de novo mutation occurring in the mother and that the mode of inheritance was consistent with autosomal sex dependent inheritance, as analyzed by the mother’s and the three children’s genetic properties.

The *AMER1* c.3245A>G variant at codon 3245 leads to substitution of arginine for histidine, and GERP++ is predicted to be conserved, suggesting that this variant may be clinically significant. This variant was not found in several databases: the Thousand Genomes Project, ESP6500, ExAC, GnomAD, and GnomAD-EAS. There are no reports of pathogenicity associated with this variant. According to the Sequence Variant Interpretation Guidelines, c.3245A>G is categorized as a variant of undetermined significance (PM2 + BP1). The *KCNE1* c.238G>C variant is a substitution of valine by isoleucine at codon 238, which is predicted to be conserved by GERP++, suggesting that a mutation at this site may be clinically significant. This variant has low frequency in the ESP, Thousand Genomes, EXAC, GnomAD, and GnomAD-EAS databases, and its pathogenicity has been reported. According to the Sequence Variant Interpretation Guidelines, c.238G>C is categorized as a variant of undetermined significance (PM1 + PM2 + PP2). Disorders associated with the AMER1 gene and the *KCNE1* gene can have a cardiac dysplasia phenotype, so variants in the *AMER1* gene and variants in the *KCNE1* gene in this family line may be responsible for their CHD.

## 4. Discussion and conclusion

In the Chinese family with preexisting heart disease that we studied here, the first proband presented with arterial ductus arteriosus and left-to-right shunting at the arterial level. By whole-exome sequencing, we identified variants in 4 genes in the family line. Those that were analyzed to fit the pattern of disease inheritance were a new hemizygous missense variant, c.3245A>G (p.His1082Arg), found in the *AMER1* gene and a new heterozygous mutant, c.238G>C (p.Val80Leu), found in the *KCNE1* gene.

**Figure 4.**
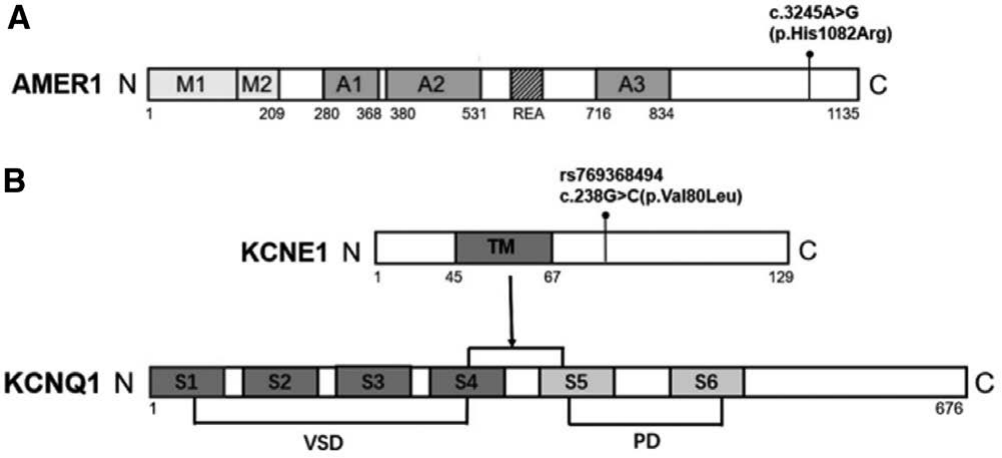
Schematic representation of the structural domains of AMER1 and KCNE1 proteins and the locations of the mutation sites in the structural domains of the proteins identified by whole-exome sequencing in this lineage. (A) AMER1 contains 3 APC-binding domains, A1–A3, that have no obvious sequence similarity to each other, 2 N-terminal phosphatidylinositol (4,5) bisphosphate-binding domains (M1 and M2), which can take up APC from the microtubule to the plasma membrane and are membrane-localized structural domains, and an arginine–glutamate–alanine (REA) motif that interacts directly with armadillo repeats of β-connexin and negatively regulates Wnt signaling. (B) KCNE1 is a single-transmembrane protein that regulates the voltage-gated potassium channel KCNQ1 by slowing its activation and enhancing its conductance. Upon activation, the positively charged voltage sensor of the VSD senses changes in membrane potential and moves outward within the membrane, opening the pore through VSD-PD coupling. The transmembrane structural domain (TM) of KCNE1 slows the activation of the channel by connecting the voltage sensor to the S4–S5 junction of the pore structural domain and restricting its movement.

*AMER1* is a potential dose-sensitive candidate gene for congenital cardiac anomalies.^[16]^ The AMER1 protein contains 3 APC-binding domains (A1–A3), 2 membrane-localized structural domains (M1 and M2) that can absorb APCs from microtubules to the plasma membrane, and a structural domain consisting of a repetitive arginine–glutamic acid–alanine motif that directly interacts with the armadillo repeats of β-conjugated proteins and is a negative regulator of Wnt signaling during development(Fig. 4A).^[17]^ Striated osteomalacia with craniosclerosis caused by variants in the *AMER1* gene can present with a congenital cardiac dysplasia phenotype in some patients.^[18]^ Other authors have found an increase in the number of copies of the *AMER1* gene in a male patient with mental retardation, congenital heart malformation, and obesity.^[16]^ *AMER1* can block typical Wnt signaling by inducing proteasomal degradation of β-conjugated proteins, and studies in zebrafish and *Xenopus laevis* have also demonstrated an inhibitory role for *AMER1* in Wnt signaling.^[19]^ Disruption of Wnt signaling is associated with congenital heart defects in animal models.^[20]^ These findings emphasize the importance of the AMER1 protein for cardiovascular development. Thus, it is possible that variants in *AMER1* contribute to CHD in humans. The *AMER1* gene variant site identified in this study is not within a known important structural domain, and its effect on protein function needs to be further investigated. The product encoded by the *KCNE1* gene is the 13th subunit of the potassium channel (Mink protein), a single-transmembrane protein that regulates the voltage-gated potassium channel KCNQ1 by slowing activation and enhancing channel conductance. The KCNQ channel contains a voltage-sensitive structural domain (VSD) and a pore structural domain. Upon activation, the positively charged voltage sensor of the VSD senses changes in membrane potential and moves outward within the membrane, opening the pore through VSD-pore structural domain coupling. The transmembrane structural domain of KCNE1 slows channel activation by connecting the voltage sensor to the S4–S5 connector of the pore structural domain and restricting its movement(Fig. 4B).^[15]^ Potassium ion channels are distributed in large numbers across the myocardial cell membrane, and the changes in their currents are closely related to myocardial cell membrane excitability. Many patients with malignant arrhythmias, such as nonfamilial arrhythmias and Jervell syndrome, have mutations in the *KCNE1* gene.^[21]^ Therefore, variations in the *KCNE1* gene may cause arrhythmias by affecting potassium ion channels, which can lead to heart disease. Further studies are needed to identify the unknown structural domains of the KCNE1 protein.

In conclusion, the novel hemizygous missense variant c.3245A>G (p.His1082Arg) in the *AMER1* gene and the de novo mutation c.238G>C (p.Val80Leu) in the *KCNE1* gene that we identified in this lineage may be the causative variants of their CHD. It is unclear how either variation contributes to the prevalent heart disease phenotype. Therefore, functional experiments in vitro and in vivo are needed to elucidate the exact mechanism linking these missense variants to their heart disease, and studies with larger sample sizes are needed to identify more cases of prevalent heart disease associated with *AMER1* and *KCNE1* variants.

Several other genetic variant loci that we identified in this whole-exome sequencing result were analyzed in this family line and did not fit the pattern of their CHD inheritance pattern. However, variants of the *PUF60* and *MED13L* genes cause disorders that can include phenotypes associated with CHD: *PUF60* gene-associated disorders such as Verheij syndrome and prenatal anomalies, and *MED13L* gene-associated disorders such as anomalous type 1 of the right great artery, mental retardation and typical facial features with or without heart defects, among others. It is possible that interactions between these genes or other factors play a role in these patients, so these mutation loci could be further validated in other family lines or by expanding the sample size to validate them in the population. The findings of such studies would further expand the genetic spectrum of prevalent heart disease.

## Acknowledgments

The authors would like to acknowledge all of the patients who participated in our study.

## Author contributions

**Conceptualization:** Caiyan An.

**Data curation:** Li Chang, Renhui Ji.

**Methodology:** Li Chang, Renhui Ji, Caiyan An.

**Writing – original draft:** Li Chang.

**Writing – review & editing:** Li Chang, Caiyan An.**Resources:** Rina Sa, Jiletu Huge.
